# PVP-SVM: Sequence-Based Prediction of Phage Virion Proteins Using a Support Vector Machine

**DOI:** 10.3389/fmicb.2018.00476

**Published:** 2018-03-16

**Authors:** Balachandran Manavalan, Tae H. Shin, Gwang Lee

**Affiliations:** ^1^Department of Physiology, Ajou University School of Medicine, Suwon, South Korea; ^2^Institute of Molecular Science and Technology, Ajou University, Suwon, South Korea

**Keywords:** bacteriophage virion proteins, feature selection, hybrid features, machine learning, support vector machine

## Abstract

Accurately identifying bacteriophage virion proteins from uncharacterized sequences is important to understand interactions between the phage and its host bacteria in order to develop new antibacterial drugs. However, identification of such proteins using experimental techniques is expensive and often time consuming; hence, development of an efficient computational algorithm for the prediction of phage virion proteins (PVPs) prior to *in vitro* experimentation is needed. Here, we describe a support vector machine (SVM)-based PVP predictor, called PVP-SVM, which was trained with 136 optimal features. A feature selection protocol was employed to identify the optimal features from a large set that included amino acid composition, dipeptide composition, atomic composition, physicochemical properties, and chain-transition-distribution. PVP-SVM achieved an accuracy of 0.870 during leave-one-out cross-validation, which was 6% higher than control SVM predictors trained with all features, indicating the efficiency of the feature selection method. Furthermore, PVP-SVM displayed superior performance compared to the currently available method, PVPred, and two other machine-learning methods developed in this study when objectively evaluated with an independent dataset. For the convenience of the scientific community, a user-friendly and publicly accessible web server has been established at www.thegleelab.org/PVP-SVM/PVP-SVM.html.

## Introduction

Bacteriophages, also known as phages, are viruses that can infect and replicate in bacteria, and are found wherever bacteria survive. The phage virion is composed of proteins that encapsulate either DNA or RNA, which binds to bacterial surface and injects its genetic materials into the specific host bacteria. In lytic cycle, phage genes are expressed for proteins that poke hole in the cell membrane, which makes cell expand and burst. Subsequently, released phages from cell bursting spread and infects other host cells. Identification of phage virion proteins (PVPs) is important for understanding the relationship between phage and host bacteria and also development of novel antibacterial drugs or antibiotics (Lekunberri et al., 2017). For instance, phage encoded proteins including endolysins, exopolysaccharidases, and holins have been proven as promising antibacterial products (Drulis-Kawa et al., 2012). Experimental methods, including mass spectrometry, sodium dodecyl sulfate polyacrylamide gel electrophoresis, and protein arrays (Lavigne et al., 2009; Yuan and Gao, 2016; Jara-Acevedo et al., 2018) have been used to identify PVPs. However, these methods are expensive and often time-consuming. Therefore, computational methods to predict PVPs prior to *in vitro* experimentation are needed. It is difficult to predict the function of PVPs from sequence information because of relatively limited experimental data. However, machine-learning (ML) approaches have been successfully applied to several similar biological problems. Therefore, it may be possible to predict the functions of phage proteins using ML.

To this end, Seguritan et al., developed the first method to classify viral structure proteins using an artificial neural network, using amino acid composition (AAC) and protein isoelectric points as input features (Seguritan et al., 2012). Later, Feng et al., developed a naïve Bayesian method, with an algorithm utilizing AAC and dipeptide composition (DPC) as input features (Feng et al., 2013b). Subsequently, Ding et al., developed a support vector machine (SVM)-based prediction model called PVPred. In this method, analysis of variance was applied to select important features from g-gap DPC (Ding et al., 2014). Recently, Zhang et al., developed a random forest (RF)-based ensemble method to distinguish PVPs and non-PVPs (Zhang et al., 2015). PVPred is the only existing publicly available method that was developed using the same dataset as our method. Although the existing methods have specific advantages in PVPs prediction, it remains necessary to improve the accuracy and transferability of the prediction model.

It is worth mentioning that several sequence-based features including AAC, atomic composition (ATC), chain-transition-distribution (CTD), DPC, pseudo amino acid composition and amino acid pair, and several feature selection techniques including correlation-based feature selection, ANOVA feature selection, minimum-redundancy and maximum-relevance, RF-algorithm based feature selection have been successfully applied in other protein bioinformatics studies (Wang et al., 2012, 2016; Lin et al., 2015; Qiu et al., 2016; Tang et al., 2016; Gupta et al., 2017; Manavalan and Lee, 2017; Manavalan et al., 2017; Song et al., 2017). All these studies motivated us in the development of a new model in this study. Hence, we developed a SVM-based PVP predictor called PVP-SVM, in which the optimal features were selected using a feature selection protocol that has been successfully applied to various biological problems (Manavalan and Lee, 2017). We selected the optimal features from a large set, including AAC, DPC, CTD, ATC, and PCP. In addition to SVM (i.e., PVP-SVM), we also developed RF and extremely randomized tree (ERT)-based methods. The performance of PVP-SVM was consistent in both the training and independent datasets, and was superior to the current method and the RF and ERT methods developed in this study.

## Materials and methods

### Training dataset

In this study, we utilized the dataset constructed by Ding et al., which was specifically used for studying PVPs (Ding et al., 2014). We decided to use this dataset for the following reasons: (i) it is a reliable dataset, constructed based on several filtering schemes; (ii) it is a non-redundant dataset and none of the sequences possesses pairwise sequence identity (>40%) with any other sequence. Hence, this dataset stringently excludes homologous sequences; and (iii) most importantly, it facilitates fair comparison between the current method and existing methods, which were developed using the same training dataset. Thus, the training dataset can be formulated as:

(1) $$\begin{array}{r} \text{S}\;=\;{\text{S}}^{+}\cup \;{\text{S}}^{-} \end{array}$$

where the positive subset ***S***^+^ contained 99 PVPs, the negative subset ***S***^−^ contained 208 non-PVPs, and the symbol **∪** denotes union in the set theory. Thus, *S* contained 307 samples.

### Independent dataset

We obtained PVP and non-PVP sequences from the Universal Protein Resource (UniProt) as previously described (Feng et al., 2013b; Ding et al., 2014; Zhang et al., 2015). To avoid overestimation in the prediction model, we excluded sequences that shared greater than 40% sequence identity with sequences in the training dataset. The final dataset contained 30 PVPs and 64 non-PVPs. We note that our independent dataset included Ding et al., independent dataset. The above two datasets can be downloaded from our web server.

### Input features

(i) AAC: The fractions of the 20 naturally occurring amino acid residues in a given protein sequence were calculated as follows:

(2) $$\begin{array}{r} \text{AAC}\left(\text{i}\right)\;=\;\frac{Frequency\;of\;amino\;acid\;\left(i\right)}{Length\;of\;the\;protein\;sequence} \end{array}$$

where *i* can be any of the 20 natural amino acids.

(ii) ATC: The fraction of five atom types (C, H, N, O, and S) in a given protein sequence was calculated as previously reported (Kumar et al., 2015; Manavalan et al., 2017), with a fixed length of five features.

(iii) CTD: The global composition feature encoding method CTD comprises properties such as hydrophobicity, polarity, normalized van der Waals volume, polarizability, predicted secondary structure, and solvent accessibility. It was first proposed in protein folding class prediction (Dubchak et al., 1995). Composition (C) represents the composition percentage of each group in the peptide sequence. Transition (T) represents the transition probability between two neighboring amino acids belonging to two different groups. Distribution (D) represents the position of amino acids (the first 25, 50, 75, or 100%) in each group in the protein sequence. For each qualitative property of a given sequence, C, T, and D produce 3, 3, and 15-dimension features, respectively. As a result, 7 × (3 + 3 + 15) = 147 features can be generated for seven qualitative properties.

(iv) DPC: The fractions of the 400 possible dipeptides present in a given protein sequence were calculated as follows:

(3) $$\begin{array}{r} \text{DPC}\left(\text{j}\right)\;=\;\frac{Total\;number\;of\;dipeptide\;\left(j\right)}{Total\;number\;of\;all\;possible\;dipeptides} \end{array}$$

where *j* can be any of the 400 possible dipeptides.

(v) PCP: We employed 11 representative PCP attributes of amino acids for feature extraction (polar, hydrophobic, charged, aliphatic, aromatic, positively charged, negatively charged, small, tiny, large, and peptide mass).

Note that all of the above features were in the range of [0, 1] as input for training and testing.

### The support vector machine

We employed a SVM as our classification algorithm, a well-known supervised ML method introduced in Boser et al. (1992) that has been applied to several biological problems (Wang et al., 2009; Eickholt et al., 2011; Deng et al., 2013; Cao et al., 2014; Manavalan et al., 2015). The objective of a SVM is to find the hyperplane with the largest margin to decrease the misclassification rate. Given a set of data points (input features) and an objective function associated with the data points (PVPs: 1 and non-PVPs: 0), SVM learn a function in the form of

(4) $$\begin{array}{r} y\;=\text{ sign}\left(\;\sum_{i\;=\;1}^{n}\alpha_{i}\;y_{i}\;K\left(x_{i},\;x\right)\;+\;b\right) \end{array}$$

where *y* is the predicted class associated with an input feature vector of *x*; α_*i*_ is the adjustable weight assigned to the training data point *x*_*i*_ during training by minimizing a quadratic objective function; *b* is the bias term; and *K* is the Kernel function. Therefore, *y* can be viewed as a weighted linear combination of similarities between the training data points *x*_*i*_ and the target data point *x*. Data points with positive weights in the training dataset affect the final solution and are called support vectors. SVM is especially effective when the input data are not linearly separable. *K* is required to map the input data into a higher dimensional space to identify the optimal separating hyperplane (Scholkopf and Smola, 2001). Therefore, we experimented with several common *K*s, including linear, Gaussian radial basis, and polynomial functions. The Gaussian radial basis *K* $\left(e^{\left(-\gamma \;\times {\;||x-y||}^{2}\right)};\;\gamma =\frac{1}{\sigma^{2}}\right)$ performed the best. Here, two critical parameters (γ and C) required optimization: γ controls how peaked Gaussians are centered on the support vectors, while *C* controls the trade-off between the training error and the margin size (Smola and Vapnik, 1997; Vapnik and Vapnik, 1998; Scholkopf and Smola, 2001). These two parameters were optimized using a grid search from 2^−15^–2^10^ for *C* and 2^−10^–2^10^ for γ, in log_2_ steps. In this study, we used a SVM implemented in the scikit-learn package (Pedregosa et al., 2011).

### Cross-validation and independent testing

As demonstrated in a series of studies (Feng et al., 2013a,c, 2018; Chen et al., 2014, 2017a,b), among three cross-validation methods, i.e., independent dataset test, K-fold cross-validation test and Leave-one-out cross-validation (LOOCV, also called jackknife cross validation), LOOCV is the most rigorous and objective evaluation methods. Accordingly, the jackknife test has been widely recognized and increasingly used to test the quality for various predictors. In LOOCV, each sample in the training dataset is in turn singled out as an independent test sample and all the rule parameters are calculated without including the one being identified. We performed LOOCV on the training dataset and the trained model was tested on the independent dataset to confirm the generality of the developed method.

### Performance evaluation criteria

The following four metrics are commonly used in literature to measure the quality of binary classification (Xiong et al., 2012; Li et al., 2015): sensitivity, specificity, accuracy and Matthews' correlation coefficient (MCC), which are expressed as

(5) $$\{\begin{array}{c} Sensitivity\;=\;\frac{TP}{TP\;+\;FN}\\ Specificity\;=\;\frac{TN}{TN\;+\;FP}\\ Accuracy\;=\;\frac{TP\;+\;TN}{TP\;+\;FP\;+\;TN\;+\;FN}\\ MCC\;=\;\frac{TP\;\times \;TN\;-\;FP\;\times \;FN}{\sqrt{\left(TP\;+\;FP)(TP\;+\;FN)(TN\;+\;FP)(TN\;+\;FN\right)}} \end{array}$$

where *TP* is the number of PVPs predicted to be PVPs; *TN* is number of non-PVPs predicted to be non-PVP; *FP* is the number of non-PVPs predicted to be PVP; and *FN* is the number of PVPs predicted to be non-PVP.

To further evaluate the performance of the classifier, we employed a receiver operating characteristic (ROC) curve. The ROC curve was plotted with the false positive rate as the x-axis and true positive rate as the y-axis by varying the thresholds. The area under the curve (AUC) was used for model evaluation, with higher AUC values corresponding to better performance of the classifier.

## Results

### Framework of the proposed predictor

**Figure 1:**
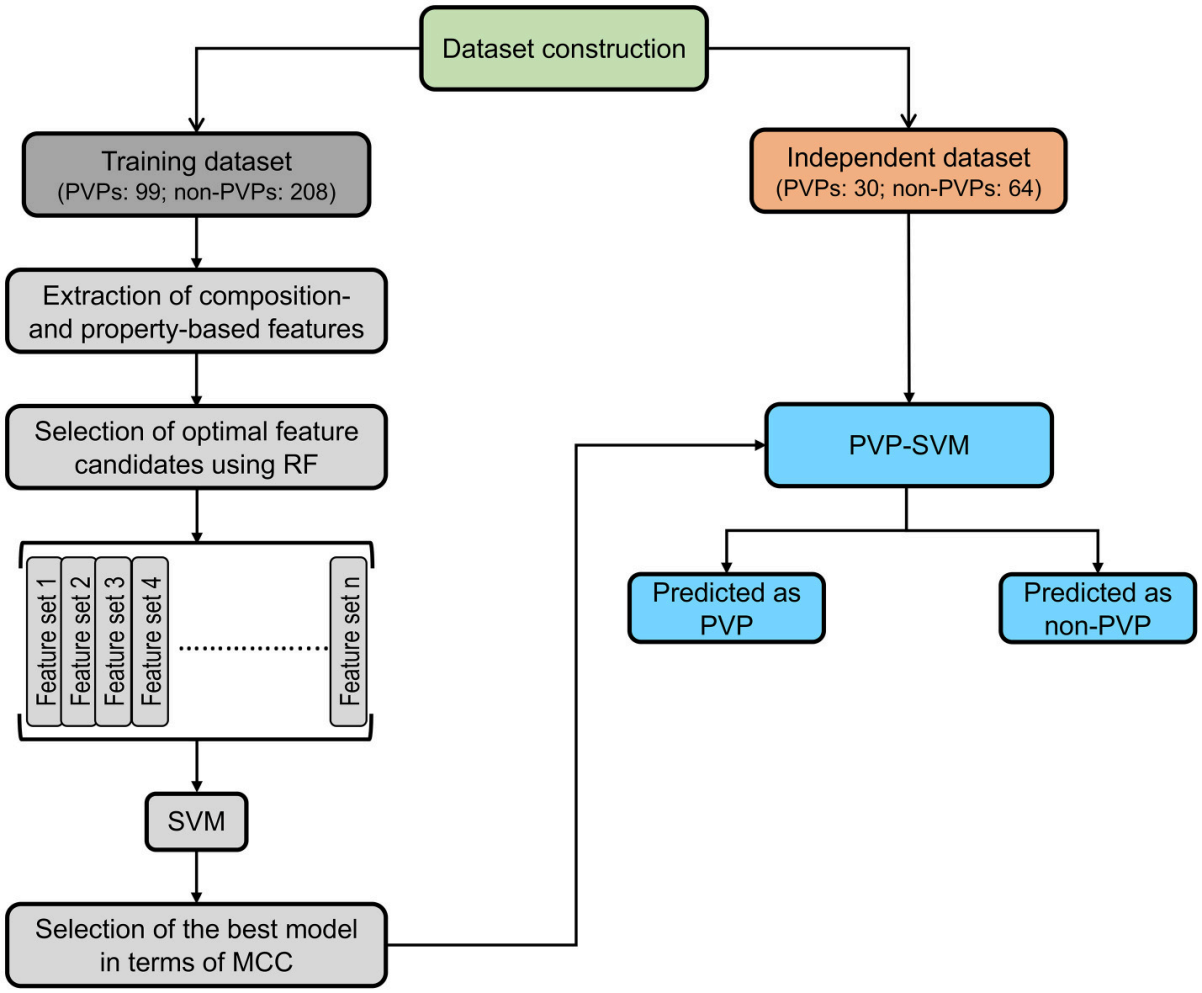
PVP-SVM development consisted of four steps: (i) dataset construction; (ii) feature extraction; (iii) development of the prediction model; and (iv) selection of the best model and construction of PVP-SVM.

Figure 1 illustrates the overall framework of the PVP-SVM method. It consisted of four steps: (i) construction of the training and independent datasets; (ii) extraction of various features from the primary sequences, including AAC, ATC, CTD, DPC, and PCP; (iii) generation of 25 different feature sets based on feature importance scores (FIS) computed using the RF algorithm. These different sets were inputted to the SVM to develop their respective prediction models; and (iv) the model producing the best performance in terms of MCC was considered the final model, and the corresponding feature set was considered the optimal feature set.

### Feature selection protocol

Generally, high dimensional features can contain a higher degree of irrelevant and redundant information that may greatly degrade the performance of ML algorithms. Therefore, it is necessary to apply a feature selection protocol to filter the redundant features and increase prediction efficiency (Wang et al., 2012; Zheng et al., 2012; Manavalan et al., 2014; Manavalan and Lee, 2017; Song et al., 2017). Previously, Manavalan and Lee applied a systematic feature selection protocol and developed a novel quality assessment method called SVMQA (Manavalan and Lee, 2017), which was the best method in CASP12 blind prediction experiments (Elofsson et al., 2017; Kryshtafovych et al., 2017). We applied a similar protocol in our recent studies, including cell-penetrating peptide and DNase I hypersensitivity predictions (Manavalan et al., 2018). Interestingly, this protocol significantly improved the performance of our method. Therefore, we extended this approach to the current problem. The current protocol differs slightly from the published protocol in terms of parameters (*ntree* and *mtry*) used in the RF algorithm, which is mainly due to the large number of features used in this study (i.e., 26-fold more features than were used in SVMQA).

**Figure 2:**
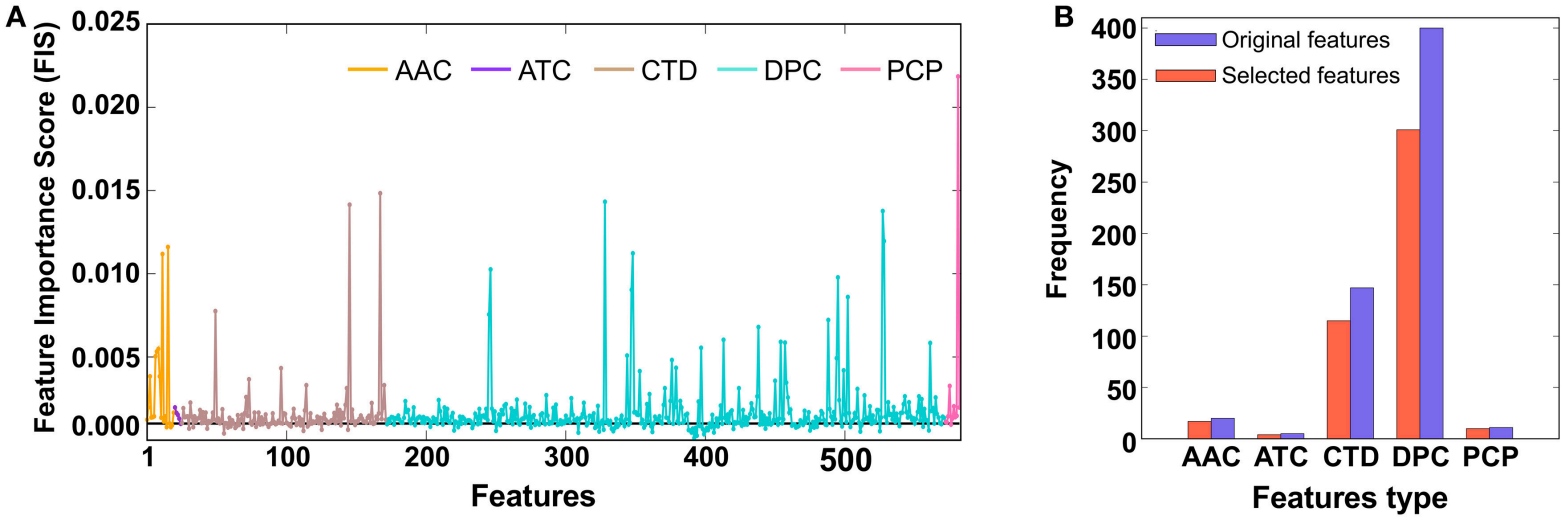
**(A)** The x- and y-axes represent each feature and its feature importance scores (FIS), respectively. We applied a FIS cutoff ≥ 0.001 and selected 477 optimal feature candidates. **(B)** Distribution of each feature type in the optimal feature candidates and original feature set.

In our study, each protein sequence was represented as 583 dimensional vectors, which was higher than the number of samples. In the first step, we applied the RF algorithm and estimated the FIS of 583 features (AAC: 20; DPC: 400; ATC: 5; PCP: 11; and CTD: 147) to distinguish PVPs and non-PVPs. A detailed description of how we computed the FIS scores of the input features has been reported previously (Manavalan et al., 2014; Manavalan and Lee, 2017). Briefly, we used all features as inputs in the RF algorithm and performed ten-fold cross-validation using the training dataset. For each round of cross-validation, we built 5,000 trees, and the number of variables at each node was chosen randomly from 1 to 100. The average FIS from all the trees are shown in Figure 2A, where most of the features had similar scores and only ~5% (FIS ≥ 0.005) contributed significantly to PVP prediction. In the second step, we applied a FIS cutoff ≥ 0.001 and selected 477 features as optimal feature candidates (Figure 2B). Subsequently, we generated 25 different sets of features from the optimal feature candidates based on an FIS cut-off (0.001 ≤ FIS ≤ 0.004, with a step size of 0.0011). Basically, we considered each set of more important features in a step-wise manner. To identify the optimal feature set, we inputted each set into the SVM separately and performed LOOCV to evaluate their performance. The prediction model that produced the best performance (i.e., the highest MCC) was considered final, and the corresponding feature set was considered optimal.

### Performance of various prediction models on the training dataset

**Figure 3:**
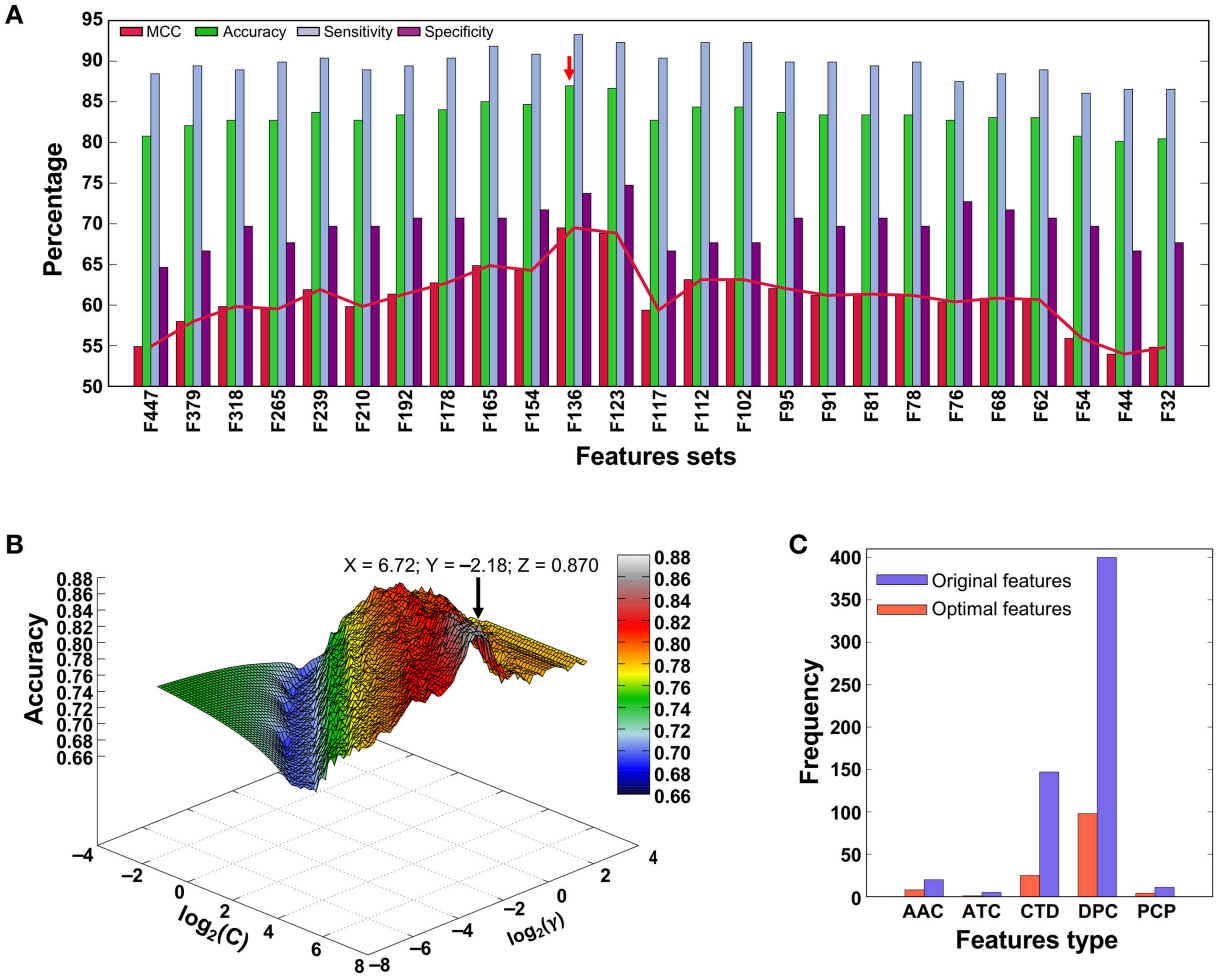
**(A)** Performance of SVM-based classifiers in distinguishing between PVPs and non-PVPs. A total of 25 classifiers were evaluated using LOOCV, and their performances in terms of MCC, accuracy, sensitivity, and specificity are shown. The red arrow denotes the final selected model. **(B)** Classification accuracy of the F136-based (final selected) model with respect to variations in parameters *C* and γ. **(C)** Distribution of each feature type in the optimal feature set (136 features) and original feature set (583 features).

Figure 3A shows the performances of the SVM model using different sets of input features, in which the MCC gradually increased with respect to the different feature sets, peaked with the F136-based model, and then gradually declined. Figure 3B shows the classification accuracy vs. parameter variation (*C* and γ) of the final F136-based model. The maximal classification accuracy was 0.870, when the parameters log_2_(*C*) and log_2_(γ) were 6.72 and −2.18, respectively, with MCC, sensitivity, and specificity values of 0.695, 0.737, and 0.933, respectively. The feature type distribution of the optimal feature set and the total features employed in this study are shown in Figure 3C. Among 136 optimal features, there were eight AAC features, one ATC feature, 25 CTD features, 98 DPC features, and four PCP features, indicating that important properties from all five compositions contributed to PVP prediction.

To demonstrate the effect of our feature selection protocol, we compared the F136-based model with the control SVM (using all features) and also an individual composition-based prediction model. As shown in Table 1, F136-based model accuracy, MCC, and area under curve (AUC) were 15–44, 6–17, and 6–11% higher, respectively, than the other models. These results demonstrate that the many redundant or uninformative features present in the original feature set were eliminated through our feature selection protocol, resulting in significant performance improvement.

**Table 1:** A comparison of the proposed predictor with the individual composition-based SVM model on training dataset.

**Methods**	**MCC**	**Accuracy**	**Sensitivity**	**Specificity**	**AUC**	***P*-value**
PVP-SVM	0.695	0.870	0.737	0.933	0.900	
SVM control	0.554	0.811	0.636	0.894	0.837	0.068
AAC	0.525	0.792	0.841	0.687	0.841	0.086
DPC	0.395	0.743	0.837	0.546	0.760	***0.00023***
CTD	0.534	0.801	0.880	0.636	0.819	***0.022***
DPC	0.478	0.782	0.889	0.556	0.812	***0.014***
ATC	0.252	0.708	0.091	1.000	0.788	***0.002***

*The first column represents the method name employed in this study. The second, the third, the fourth and the fifth respectively represent the MCC, accuracy, sensitivity, and specificity. The sixth column and the seventh represent the AUC and pairwise comparison of ROC area under curves (AUCs) between PVP-SVM and the other methods using a two-tailed t-test. A P ≤ 0.05 indicates a statistically meaningful difference between PVP-SVM and the selected method (shown in bold italic)*.

### Comparison of PVP-SVM with other ML algorithms

**Figure 4:**
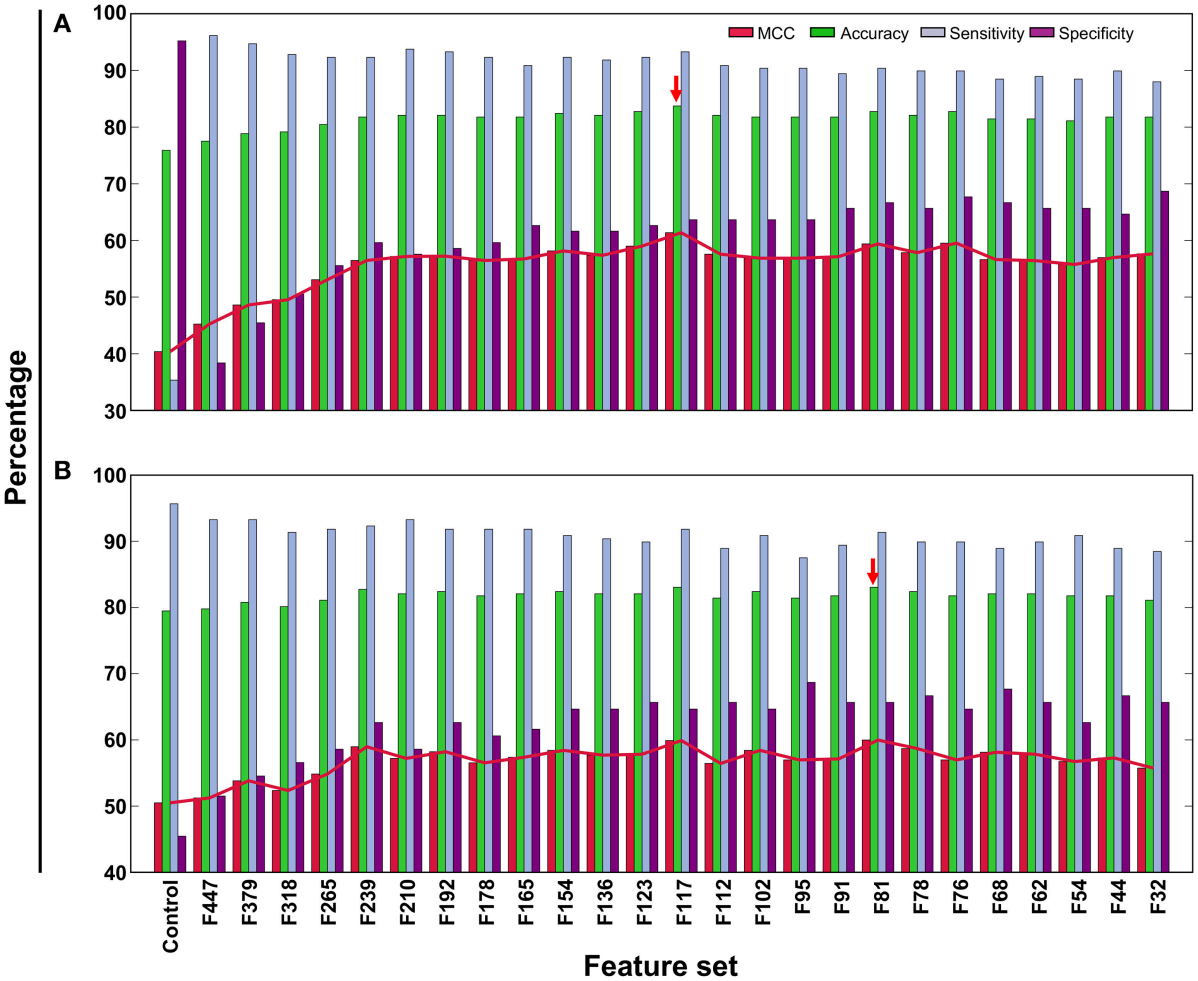
Performance of ERT- and RF-based classifiers in distinguishing between PVPs and non-PVPs. A total of 26 classifiers were evaluated using LOOCV, whose performances in terms of MCC, accuracy, sensitivity, and specificity are shown. **(A)** ERT-based performance, **(B)** RF-based performance. Red arrow denotes the final selected models for each ML method.

In addition to PVP-SVM, we also developed RF- and ERT-based models using the same feature selection protocol and training dataset (Figures 4A,B). These two methods have been described in detail in our previous study (Manavalan et al., 2017, 2018). The procedure for ML parameter optimization and final model selection was the same as for PVP-SVM. The performance of the final selected RF and ERT models was compared with PVP-SVM, as well as PVPred, which was constructed using the same training dataset. Table 2 shows that the accuracy, AUC, and MCC of PVP-SVM were 2–4, 0.1–2, and 8–9% higher, respectively, than those achieved by other methods, indicating the superiority of PVP-SVM.

**Table 2:** A comparison of the proposed predictor with other ML-based methods on training dataset.

**Methods**	**MCC**	**ACC**	**Sensitivity**	**Specificity**	**AUC**	***P*-value**
PVP-SVM	0.695	0.870	0.737	0.933	0.900	
PVPred	NA	0.850	0.758	0.894	0.899	0.974
RF	0.600	0.831	0.657	0.914	0.877	0.476
ERT	0.614	0.837	0.636	0.933	0.883	0.594

*The first column represents the method name employed in this study. The second, the third, the fourth and the fifth respectively represent the MCC, accuracy, sensitivity, and specificity. The sixth column and the seventh represent the AUC and pairwise comparison of ROC area under curves (AUCs) between PVP-SVM and the other methods using a two-tailed t-test*.

### Method performance using an independent dataset

We evaluated the performance of our three ML methods and PVPred using an independent dataset. Table 3 shows that PVP-SVM achieved the highest MCC and AUC values (0.531 and 0.844, respectively). Indeed, the corresponding metrics were 2.2–17.4% and 4.8–10.0% higher than those achieved by other methods, indicating the superiority of PVP-SVM. Specifically, PVP-SVM outperformed PVPred in all five metrics, suggesting its usefulness as an improvement to existing tools for predicting PVPs.

**Table 3:** Performance of various methods on independent dataset.

**Method**	**MCC**	**ACC**	**Sensitivity**	**Specificity**	**AUC**	***P*-value**
PVP-SVM	0.531	0.798	0.667	0.859	0.844	
ERT	0.509	0.798	0.533	0.922	0.778	0.367
RF	0.481	0.787	0.500	0.922	0.756	0.238
SVM control	0.414	0.755	0.533	0.859	0.796	0.505
PVPred	0.357	0.713	0.600	0.765	0.742	0.176

*The first column represents the method name employed in this study. The second, the third, the fourth and the fifth respectively represent the MCC, accuracy, sensitivity, and specificity. The sixth column and the seventh represent the AUC and pairwise comparison of ROC area under curves (AUCs) between PVP-SVM and the other methods using a two-tailed t-test*.

**Figure 5:**
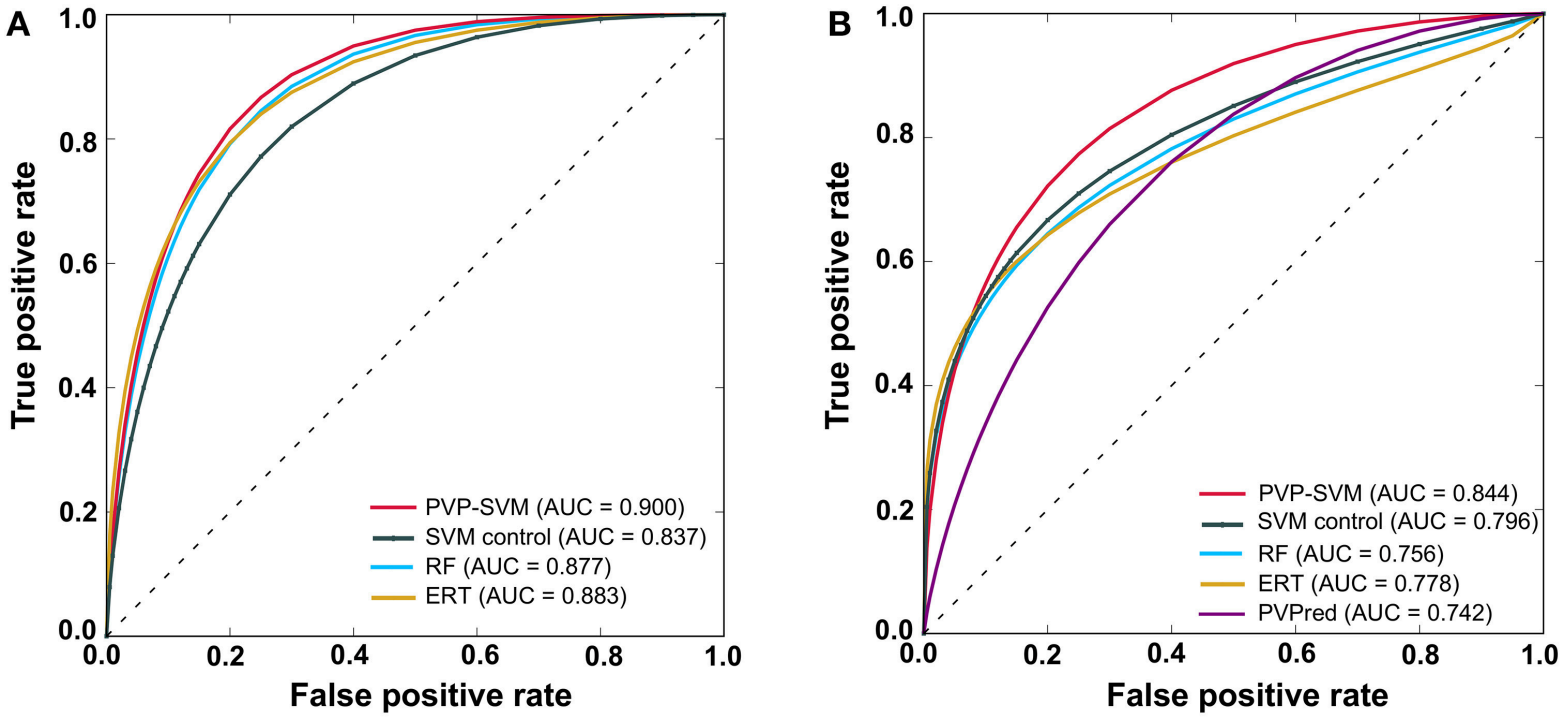
Receiver operating characteristic curves of the prediction models. **(A)** LOOCV of the training dataset. **(B)** Evaluation with an independent dataset. Higher AUC values indicate better method performance.

In general, ML-based methods are problem-specific (Zhang and Tsai, 2005). Instead of selecting a ML method arbitrarily, it is necessary to explore different ML methods on the same dataset to select the best one. Hence, we explored three most commonly used ML methods (SVM, RF, and ERT), each having its own advantages and disadvantages. The PVP-SVM method performed consistently better than other two methods both with the training and independent datasets (Figures 5A,B). Although the differences in performance between these three methods were not significant (*P* > 0.05), SVM was superior to other ML methods in PVP prediction, consistent with a previous report (Ding et al., 2014). Hence, we selected PVP-SVM as the final prediction model.

### Comparison of PVP-SVM and PVPred methodology

A detailed comparison between our method and the existing method in terms of methodology is as follows: (i) the PVPred method utilizes only g-gap dipeptides as input features, and its optimal features were determined by an analysis of variance-based feature selection protocol. However, PVP-SVM utilizes AAC, ATC, CTD, and PCP in addition to DPC, with optimal features selected based on a RF algorithm; (ii) the number of optimal features used differs between the two methods; PVP-SVM uses 136 features, while PVPred uses 160; (iii) although the same ML method was used for the two methods, the parameter optimization procedure differed, as PVP-SVM used LOOCV, while PVPred used five-fold cross-validation.

### Web server implementation

Several examples of bioinformatics tools/web servers utilized for protein function predictions have been reported in previous publications (Govindaraj et al., 2010, 2011; Manavalan et al., 2010a,b, 2011; Basith et al., 2011, 2013), and are of great practical use to researchers. To this end, an online prediction server for PVP-SVM was developed, which is freely accessible at the following link: www.thegleelab.org/PVP-SVM/PVP-SVM.html. Users can paste or upload query protein sequences in FASTA format. After submitting the input protein sequences, the results can be retrieved in a separate interface. All the curated datasets used in this study can be downloaded from the web server. PVP-SVM represents the second publicly available method for PVP prediction, and delivers a higher level of accuracy than PVPred.

## Discussion

PVPs play critical roles in adsorption between phages and their host bacteria, and are key in the development of new antibiotics. Phage-derived proteins are considered as safe and efficient antimicrobial agents due to its versatile properties, including bacteria-specific lytic mechanism, broad range of antibacterial spectrum, enhanced tissue penetration by small size, low immunogenicity, and reduced possibility for bacterial resistance (Drulis-Kawa et al., 2012). Thus, we have developed a novel computational method for predicting PVPs, called PVP-SVM. The molecular functions and biological activities of proteins can be predicted from their primary sequence (Lee et al., 2007); hence, we utilized the available PVPs sequences to develop the method.

A combination of AAC, ATC, DPC, CTD, and PCP features was used to map the protein sequences onto numeric feature vectors, which were inputted into the SVM to predict PVPs. Although AAC, CTD, and DPC features have been used previously (Feng et al., 2013b; Ding et al., 2014; Zhang et al., 2015), this is the first report including ATC and PCP. In ML-based predictions, feature selection is one of the most important steps because of redundant and non-informative features. Generally, high dimensional features contain numerous non-informative and redundant features, which affect prediction accuracy. Hence, the feature selection protocol is considered one of the most important steps in ML-based prediction (Wang et al., 2012; Manavalan et al., 2014; Manavalan and Lee, 2017; Song et al., 2017). To this end, we applied a feature selection protocol that has been proven effective in various biological applications (Manavalan and Lee, 2017; Manavalan et al., 2018), and identified the optimal features. Of those, the major contribution was from DPC (~72%), followed by CTD, AAC, PCP, and ATC, indicating that information about the fraction of amino acids as well as their local order might play a major role in predicting PVPs. A previous study demonstrated that basic amino acids (Lys and Arg) usually occur in the flanking potential cleavage site in PVPs, as their side chain flexibility is required to accommodate the change observed in the cleavage site (Coia et al., 1988; Speight et al., 1988). Interestingly, our optimal features contain these two important types of residues.

In general, if a prediction model is developed using a training dataset that contains highly homologous sequences, this method will overestimate the prediction accuracy. In this regard, Feng et al., and Ding et al., used a lower homology (<40% sequence identity) sequence dataset to develop their prediction models (Feng et al., 2013b; Ding et al., 2014). Zhang et al., developed their model using a highly homologous sequence dataset (<80% sequence identity); as a result, this method showed higher accuracy when evaluated with an independent dataset (Zhang et al., 2015). Furthermore, PVPred is the only publicly available method of the three, in the form of a web server, and was generated using the same dataset as our method. Therefore, we compared the performance of our method with PVPred only. Generally, a prediction model tends toward over-optimization in order to attain higher accuracy. Therefore, it is always necessary to evaluate the prediction model using an independent dataset, to measure the generalizability of the method (Chaudhary et al., 2016; Manavalan and Lee, 2017; Nagpal et al., 2017). Hence, we evaluated our three prediction models and PVPred on an independent dataset. Our study demonstrated that PVP-SVM consistently performed better than PVPred and the two other methods developed in this study on both datasets, indicating the greater transferability of the method.

The superior performance of PVP-SVM may be attributed to two important factors: (i) integration of previously reported features and inclusion of novel features that collectively make significant contributions to the performance; and (ii) a feature selection protocol that eliminates overlapping and redundant features. Furthermore, our approach is a general one, which is applicable to many other classification problems in structural bioinformatics. Although PVP-SVM displayed superior performance over the other methods, there is room for further improvements, including increasing the size of the training dataset based on the experimental data available in the future, incorporating novel features, and exploring different ML algorithms including stochastic gradient boosting (Xu et al., 2017) and deep learning (LeCun et al., 2015).

A user-friendly web interface has been made available, allowing researchers access to our prediction method. Indeed, this is the second method to be made publicly available, with higher accuracy than the existing method. Compared to experimental approaches, bioinformatics methods, such as PVP-SVM, represent a powerful and cost-effective approach for the proteome-wide prediction of PVPs. Therefore, PVP-SVM might be useful for large-scale PVP prediction, facilitating hypothesis-driven experimental design.

## Author contributions

BM and GL conceived and designed the experiments; BM performed the experiments; BM and TS analyzed the data; BM and GL wrote paper. All authors reviewed the manuscript and agreed to this information prior to submission.

### Conflict of interest statement

The authors declare that the research was conducted in the absence of any commercial or financial relationships that could be construed as a potential conflict of interest.
